# Reproducible computational biology experiments with SED-ML - The Simulation Experiment Description Markup Language

**DOI:** 10.1186/1752-0509-5-198

**Published:** 2011-12-15

**Authors:** Dagmar Waltemath, Richard Adams, Frank T Bergmann, Michael Hucka, Fedor Kolpakov, Andrew K Miller, Ion I Moraru, David Nickerson, Sven Sahle, Jacky L Snoep, Nicolas Le Novère

**Affiliations:** 1 Department of Systems Biology & Bioinformatics, Institute of Computer Science, University of Rostock, D-18051 Rostock, Germany; 2 Centre for Systems Biology Edinburgh, CHWaddington Building, University of Edinburgh, Edinburgh EH9 3JD, UK; 3 California Institute of Technology, 1200 East California Blvd., Pasadena, CA 91125, USA; 4 Institute of Systems Biology Ltd., Detskiy proezd 15, Novosibirsk, 630090, Russia; 5 Auckland Bioengineering Institute, The University of Auckland, Auckland, New Zealand; 6 Center for Cell Analysis and Modeling, University of Connecticut Health Center, Farmington, CT 06030, USA; 7 BIOQUANT, University of Heidelberg, Im Neuenheimer Feld 267, Heidelberg, Germany; 8 Department of Biochemistry, Stellenbosch University, Privatebag X1, Matieland 7602, South Africa; 9 Manchester Centre for Integrative Systems Biology, Manchester Interdisciplinary Biocentre, the University of Manchester, 131 Princess Street Manchester, M1 7DN, UK; 10 Molecular Cell Physiology, VU University, Amsterdam, The Netherlands; 11 EBI, Wellcome Trust Genome Campus, Hinxton, Cambridgeshire CB10 1SD, UK

## Abstract

**Background:**

The increasing use of computational simulation experiments to inform modern biological research creates new challenges to annotate, archive, share and reproduce such experiments. The recently published *Minimum Information About a Simulation Experiment *(MIASE) proposes a minimal set of information that should be provided to allow the reproduction of simulation experiments among users and software tools.

**Results:**

In this article, we present the Simulation Experiment Description Markup Language (SED-ML). SED-ML encodes in a computer-readable exchange format the information required by MIASE to enable reproduction of simulation experiments. It has been developed as a community project and it is defined in a detailed technical specification and additionally provides an XML schema. The version of SED-ML described in this publication is *Level 1 Version 1*. It covers the description of the most frequent type of simulation experiments in the area, namely time course simulations. SED-ML documents specify which models to use in an experiment, modifications to apply on the models before using them, which simulation procedures to run on each model, what analysis results to output, and how the results should be presented. These descriptions are independent of the underlying model implementation. SED-ML is a software-independent format for encoding the description of simulation experiments; it is not specific to particular simulation tools. Here, we demonstrate that with the growing software support for SED-ML we can effectively exchange executable simulation descriptions.

**Conclusions:**

With SED-ML, software can exchange simulation experiment descriptions, enabling the validation and reuse of simulation experiments in different tools. Authors of papers reporting simulation experiments can make their simulation protocols available for other scientists to reproduce the results. Because SED-ML is agnostic about exact modeling language(s) used, experiments covering models from different fields of research can be accurately described and combined.

## Background

Reproducibility of results is a basic requirement for all scientific endeavors. This is not only true for experiments in the wet lab, but also for simulations of computational biology models [1]. Reproducibility of simulations (i. e., the closeness between the results of independent simulations performed with the same methods on identical models but with a different experimental setup [1]) saves time in modeling and simulation projects. The Minimum Information About a Simulation Experiment (MIASE, [1]) is a reporting guideline describing the minimal set of information that must be provided to make the description of a simulation experiment available to others. It includes the list of models to use and their modifications, all the simulation procedures to apply and in which order, the processing of the raw numerical results, and the description of the final output. MIASE is part of MIBBI [2], a project aiming at federating Minimum Information guidelines (MIs) in the life sciences. MIs are standards that specify which information should be provided as a minimum to ensure that published results of a given type can be understood, reused, and reproduced. MI standards focus on the information to be provided, but do not specify under which form it must be provided.

Different data formats have been developed to support the encoding of computational models of biological systems. Such model representation formats include, for example, SBML [3], CellML [4] and NeuroML [5]. However, while these formats are widely accepted and used to describe model structure, they do not cover the description of simulation, or analyses performed with the models. To address this need, we created the Simulation Experiment Description Markup Language (SED-ML, http://sed-ml.org/), an XML-based format for the encoding of simulation experiments performed on a set of computational models. Here, we describe SED-ML and its development process as a community project in detail.

## Results

**Figure 1:**
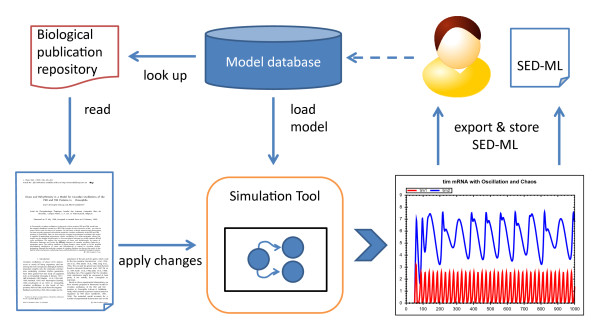
**The role of SED-ML in a modeler's simulation workflow**. To reuse an existing model in a simulation tool, end-users (1) need to retrieve the model from a repository, (2) read the reference publication to apply the correct pre-processing to the model, and then (3) configure the simulation tool. SED-ML improves the situation by allowing to encode all these steps computationally. A user thus can store, archive, and export simulation experiment descriptions for his own records or for sharing with fellow researchers. Two arrows are used from "time plot" to "SED-ML" to depict that the SED-ML model will either be exported and stored as SED-ML, or shared with a fellow researcher. The dashed line is the starting point of the figure, with a researcher aiming at reusing a model from a repository in a simulation.

SED-ML encodes the description of simulation experiments in XML, in an exchangeable, reusable manner. Figure 1 shows how SED-ML could fit into a modeler's simulation workflow: Ideally, model authors will provide SED-ML files together with their publications, describing how to reproduce the presented simulation results. End-users will then be able to download models together with applicable simulation setups, enabling them to directly run the simulation in a simulation software. End-users might in addition share their own simulation experiment descriptions by exporting SED-ML from their simulation tool.

**Figure 2:**
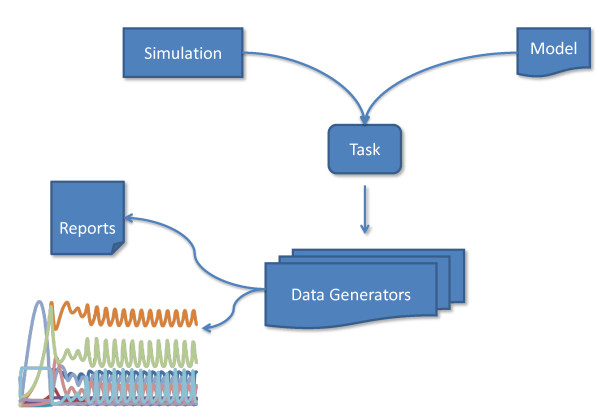
**Main SED-ML elements**. High level overview of the relations between the five major elements of a SED-ML document. Pairs of model and simulation elements are used in tasks. The dataGenerators allow to define the post-processing of simulation data to define the desired output (plots or reports).

SED-ML is built of five main descriptive elements: (1) the models used in the experiment; (2) the simulation algorithms and their configurations; (3) the combination of algorithm and model into a numerical experiment; (4) post-processing of results; (5) and output of results. The relations between these elements are illustrated in Figure 2.

### (1) Model elements

define the identity and location of the model(s) to be simulated and specify the model's native encoding format. The location is to be given as a Uniform Resource Identifier (URI), which enables software interpreting SED-ML to retrieve the model. In case of a relative URI, the base is the location of the referring SED-ML file. To share model and simulation descriptions together, we advise the use of the SED-ML archive format, described in the specification. To link the SED-ML file to remote model descriptions, we recommend using persistent, consistent and accessible model resources. Persistent model resources include, for instance, repositories or databases having a MIRIAM URI [6]. We have restricted SED-ML to model encodings in XML-based languages (such as SBML, CellML, or NeuroML). In order to improve interoperability, the particular language a model is encoded in should be specified using one of the predefined SED-ML language Uniform Resource Name (URN); the list is available from the SED-ML website. Using URNs, one can specify a language precisely (e. g., "SBML Level 3, Version 1") or generically (e. g., "CellML (generic)"). Further languages can be registered via the SED-ML website.

In addition to defining the source model's location and encoding, SED-ML model elements can also list changes to be applied to a model before simulation. Such changes could be altering attribute values (e. g., a parameter value in an SBML model or the initial_value of a CellML variable) or changing the model structure. Attribute values may undergo a simple substitution or more complex calculation using content MathML 2.0 [7]. The model structure may be changed by adding or removing XML elements. XPath [8] expressions identify the target XML to which a change should be applied, thereby identifying model entities required for manipulation in SED-ML.

### (2) Simulation elements

define the simulation algorithms to be used in the experiment and their configuration. Simulation algorithms are specified using terms from the Kinetic Simulation Algorithm Ontology (KiSAO, http://biomodels.net/kisao/, [9]). KiSAO classifies and characterizes kinetic simulation algorithms, such as those commonly used in systems biology. Furthermore, configuration details of the simulation can be described in SED-ML, such as the start and end times, or the number of time points to output. The current implementation supports the description of time course simulation setups. Extensions towards further experiment types are already being discussed and will be available in the next versions, including the description of steady-state analyses and nested simulations, such as parameter scans.

### (3) Task elements

apply a particular simulation algorithm to a specific model. Because simulations and models are described independently, they can be combined in diverse ways. For example, the behavior of one model can be tested with a deterministic and a stochastic simulation algorithm, or a simulation can be applied to different versions of a model with varying parameterization (or other arbitrary model changes applied to the SED-ML model element)

### (4) Data Generator elements

define transformations of raw simulation output generated by a task into the desired numerical form. For example, the simulation output might need normalization or scaling before output. Data generators can simply be references to a model variable, but may also be defined through complex mathematical expressions encoded using content MathML. Some variables used in an experiment are not explicitly defined in the model, but may be implicitly contained in it and therefore not addressable using XPath. The 'time' variable in SBML is a common example. To allow SED-ML to refer to such variables in a standard way, the notion of implicit variables has been incorporated into SED-ML. These so-called symbols are defined following the idea of MIRIAM URNs and using the aforementioned SED-ML URN scheme. To refer to the definition of SBML 'time' from a SED-ML file, for example, the URN is urn:sedml:symbol:time. The list of predefined symbols is available from the SED-ML website. From that source, a mapping of SED-ML symbols onto possibly existing concepts in the individual languages supported by SED-ML is provided.

### (5) Output elements

describe how numerical data from the data generators are grouped together. In SED-ML Level 1 Version 1, one can relate two data streams or three data streams, allowing to generate 2D and 3D plots, or provide all the data streams as a set of unrelated arrays.

SED-ML documents can contain zero or more instances of the element types described above. A document describing several simulation experiments in a single file enables multiple simulations on the same set of models; for example, the output obtained from different simulation algorithms could be compared. Alternatively, a SED-ML document linking to several models enables the encoding of experiments to determine the influence of changes to models on the simulation output. Moreover, a SED-ML document describing several outputs provides the user with different views of the simulation results. Future versions of SED-ML may also allow the encoding of chained simulations (where several simulations are to be performed in a predefined order and results from one simulation are used to initialize a subsequent simulation).

All SED-ML elements can be complemented with human-readable notes written in XHTML, and machinereadable annotations. Furthermore annotations enable users to extend SED-ML to cover simulation and analysis procedures that are not (yet) part of the core language. The re-use of other standardized formats inside SED-ML annotations is encouraged; for example, simulation outputs can be annotated with terms from the Terminology for the Description of Dynamics (TEDDY, http://www.ebi.ac.uk/compneur-srv/teddy/, [9]). When annotating SED-ML elements with meta-information, MIRIAM URIs [6] should be used. In addition to providing the data type (e. g., PubMed) and the particular data entry inside that data type (e. g., 10415827), the annotation should be related to the annotated element using the standardized http://BioModels.net qualifiers. The list of qualifiers, as well as further information about their usage, is available from http://biomodels.net/qualifiers/.

**Figure 3:**
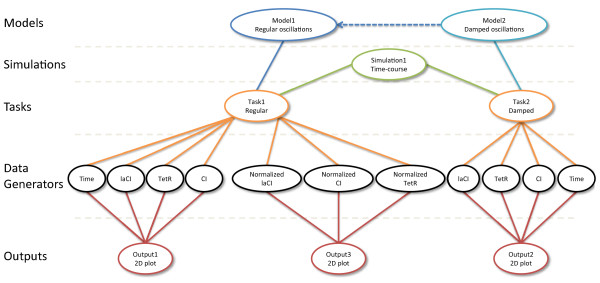
**SED-ML document content**. Graphical representation of the contents of the SED-ML document describing simulation experiments performed on the Repressilator model [25]. Three outputs are specified. Output 1 (left) displays a plot of levels of three proteins against time, showing regular oscillations. Output 2 (right) produces a plot of the same three protein levels against time after altering the model to produce damped oscillations. Output 3 (center) describes a plot of a simulation of regular oscillations, but after normalizing results and plotting the normalized amounts of each protein against each other. The simulations are described in more detail in Section 1.1 of the SED-ML specification [24], and the SED-ML document is available from the additional file 1.

Figure 3 shows a graphical representation of a SED-ML file, illustrating how the components described above can be used. In this example, a reference model, model1, is obtained from BioModels Database, while model2 is derived from the reference model by altering a parameter's value. Each model is simulated using identical solver configurations, and various outputs are derived from the main results.

**Figure 4:**
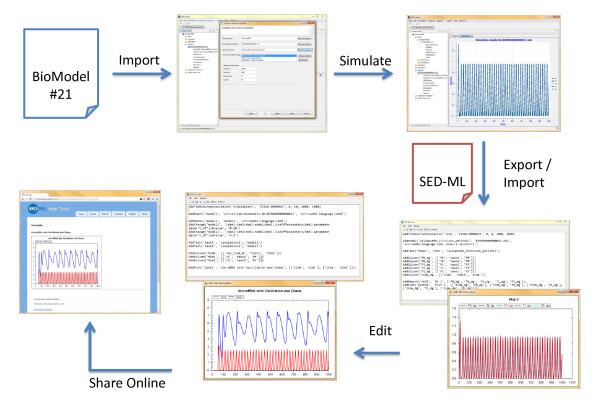
**SED-ML demonstration**. SED-ML facilitates the exchange of simulation experiments for a number of modeling languages and supports a number of existing simulators. In this sample setup, the SBML model file *BIOMD0000000021 *(available from BioModels Database) was loaded into SBSIVisual, a simulation was created and then exported into the SED-ML Script editor (where the simulation can be reproduced). There the simulation was edited to carry out a simulation on a changed model. Finally, the resulting SED-ML experiment was saved as an archive and uploaded to the SED-ML Web Tools, where it was run to reproduce the simulation experiment.

Clearly, the interpretation and execution of SED-ML files will require software support. This is increasingly available, both in the form of application support for end-users wishing to execute simulations encoded in SED-ML, and as software libraries to facilitate the uptake of SED-ML support amongst application developers. To demonstrate the capability of SED-ML to facilitate the exchange of simulation experiment descriptions, we chose several freely available independent applications that support SED-ML: SED-ML Web tools (http://sysbioapps.dyndns.org/SED-ML_Web_Tools/), libSedMLScript (http://libsedml.sourceforge.net/) and SBSIVisual (http://www.sbsi.ed.ac.uk/). In SBSIVisual, we ran a simulation of a simple Circadian clock model [10] to produce oscillating behavior, and exported the simulation configuration in SED-ML format. We then edited this SED-ML file using libSedMLScript to describe how the model can produce chaotic behavior, and uploaded it to SED-ML Web Tools to execute and display both simulations. The workflow is shown in Figure 4.

## Discussion

In this article, we describe SED-ML, a language to encode procedures performed during computational simulation experiments, and its development process. The first version of SED-ML focuses on encoding uniform time-series experiments, since these are the most widely-used types of numerical model analysis in systems biology. They generally only require a model, and no additional resources such as experimental data.

We expect to extend future versions of SED-ML to include references to experimental data, as the standards and availability of relevant data develop. This is an essential first step towards encoding more complicated experiments such as nested simulations, parameter sweeps, parameter estimation, and sensitivity analysis. The limited scope of SED-ML Level 1 Version 1 lays a firm foundation from which to proceed, and any issues arising from its implementation can be dealt with better at an early stage. Moreover, an early release of a subset of the anticipated future functionality, with widespread community support, fosters participation and uptake amongst the modeling communities targeted by SED-ML.

As SED-ML evolves to describe more complex simulation experiments it will be increasingly useful to link models, simulation descriptions, and experimental data together in a machine-readable way. SED-ML describes the computational steps needed to reproduce particular results of a computational simulation, but it does not encode the simulation results themselves. The latter could be achieved, for instance, by the *Numerical Markup Language *(NuML, http://code.google.com/p/numl/). NuML initially had been part of the *Systems Biology Result Markup Language *(SBRML, [11]), a format to link a model with simulated and experimental datasets. SBRML used a free text 'Software' element to define the software tool, version and algorithm used to generate results. In addition, it will now provide the possibility to point towards a SED-ML file from the SBRML 'Method' element. Both SBRML and SED-ML will use NuML to store lists of numbers, either results or datasets.

SED-ML is agnostic about the underlying model representation formats and the software tool that gave rise to the experiment. The model variables that a SED-ML model needs to be aware of are addressed directly by XPath. SED-ML can thus encode simulation experiments involving models in different formats. Currently SED-ML is restricted to models encoded in XML-based formats. However, we envision that MIASE-compliant models may not always be XML-based and SED-ML should endeavor to address those formats in the future. Whilst many applications are tied to a particular modeling language, the increasing provision of simulation tools as web services [12] would enable a computational workflow to execute such a SED-ML description. The goals of SED-ML closely align with those of the earlier RDF-based CellML Simulation and Graphing Metadata specifications [13] and in the interests of developing a common standard, development of those metadata specifications has been migrated to SED-ML.

While the contributors to the development of the language are primarily from the systems biology community, there is no reason why SED-ML could not be used in other domains that use computational simulation, such as environmental or agricultural modeling, neuroscience or pharmacometrics. Various communities, working on biological model representations, have already committed to the use and support of SED-ML, including SBML, CellML, and the Virtual Cell. Promotion of SED-ML in other realms of science and model representation communities (e. g., ISML, NeuroML, NineML, SimileXML ...) is an ongoing focus. Some of these communities have implemented software support for SED-ML in different tools, including SED-ML validators and a SED-ML visual editor. An up-to-date list is available at the SED-ML website.

The model changes specified in a SED-ML file result in implicit new models. These new models are only instantiated by the simulation environment interpreting the SED-ML file. This important feature of SED-ML allows the exploration of many different model structures to be stored in a compact way. Other methods have been proposed in the past, such as XML diff and patch [14]. This allows not only to change the parametrization of a model by changing the value of an XML attribute, but also to change the structure of the model by adding or removing XML construct. If a user then decides that the result of such changes is a new model, he may choose not to export a simulation description with that set of changes, but to store the modified version as a new model and use it as such in the simulation description. SED-ML is intended to be used by simulation software, as an export/import format. Therefore, the changes that are applicable to a model have to be specifiable within the software tool. As such, the software is responsible for only allowing valid model updates - and also for correctly translating them into SED-ML concepts. SED-ML itself does not restrict the changes that can be applied to the models mentioned in a SED-ML file.

A number of software libraries have already been made available in C++, Java and .NET. We briefly describe a few of them in the following paragraphs.

**libSedML **http://libsedml.sf.net/ is a set of .NET libraries for supporting SED-ML. The core library libSedML supports reading, validating and writing of SED-ML descriptions, along with all necessary utility functions for resolving models and XPath expressions. Two additional libraries are included: libSedML-Runner, which allows to schedule and execute simulation experiments encoded in SED-ML files using either RoadRunner (http://roadrunner.sf.net/, [15]) or a variety of simulators exposed through the Systems Biology Workbench (SBW, [16]), such as iBioSim [17] and COPASI [18]. A third library, libSedMLScript, provides a script-based language for defining SED-ML experiments.

**jlibsedml **(http://sourceforge.net/projects/jlibsedml/) is a Java library for creating, manipulating, validating and working with SED-ML documents. It provides support for retrieval and pre-processing of models, by application of XPath expressions, and also post-processing of raw simulation results as specified by SED-ML dataGenerator elements. The jlibsedml application programming interface (API) follows a similar organization to that of libSBML [19], a successful and popular library for manipulation of SBML documents.

**SProS **(the SED-ML Processing Service) is an API described in interface definition language (IDL) for creating, reading and manipulating SED-ML documents, and so can be used by multiple software packages. The CellML API [20] provides an implementation of SProS. Future versions of SProS will also provide support for running simulations described in SED-ML and involving CellML models (using the simulation facilities already present in the CellML API).

**Figure 5:**
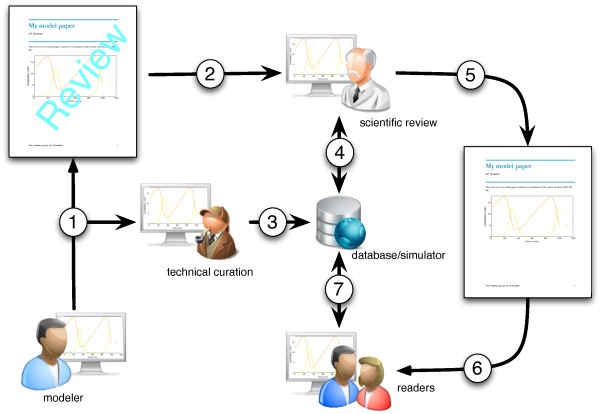
**SED-ML, publications and model databases**. Many Figures in journal publications cannot easily be reproduced by the curators of model databases and later end-users. The Figure shows how SED-ML can help model curators in reproducing simulations submitted together with a manuscript. A workflow for the publication process would involve the following steps: 1, a researcher submits a manuscript with a mathematical model to a scientific journal; 2, the manuscript is sent out for scientific review; the researcher can submit his model directly to a model database such as BioModels Database or JWS Online, either concurrently with step 1 or via the journal office. 3, Model curators perform a technical curation of the model, (i.e. check whether the model description is complete, whether the model can be simulated and whether the results shown in the manuscript can be reproduced); 4, if the model passes the technical curation it could be made available for the scientific reviewer on a secure site (as it is the case for instance with JWS Online); 5, after scientific review the manuscript might become acceptable and published; 6, after which readers can access the manuscript; and 7, the model is moved to the public database, and is accessible for simulation. SED-ML would greatly facilitate steps 3, 4, and 7."

We see an important role for SED-ML in the publication workflow, and in the enrichment it can bring to manuscripts containing mathematical models. Many journals currently require that models described in a manuscript be made available in electronic form, often in SBML, but software-specific formats are also accepted. Although reviewers would ideally test these models during the review process, this is often not done, perhaps due to time pressure or unfamiliarity with modeling software. As a consequence, many figures that show simulation results cannot be reproduced by the models linked to the manuscript, resulting in a labor-intensive curation step for model repositories, such as BioModels Database [21] and JWS Online model repository [22]. To aid in the reviewing process and prevent discrepancies between manuscript and model, JWS Online, to give one example, has set up a model reviewing workflow with a number of journals. The workflow consists of an initial check by the curators to reproduce simulations in a submitted manuscript. SED-ML will make this workflow significantly easier. Ideally, modelers would provide SED-ML scripts with their manuscript submission, these scripts can be run directly by the curator and make the curation job much easier. If the SED-ML scripts are not provided upon model submission they are generated by the curator and made available to the manuscript reviewer. The script loads the respective model and returns the model simulation. A SED-ML script can be linked to each simulation figure in the manuscript. This publication workflow is shown in Figure 5.

SED-ML Level 1 Version 1 provides a foundation for storing simulation experiment descriptions. It is designed to be easily extensible through the definition of further simulation (and analysis) types. The community is already discussing several such extensions, and in particular to cover nested simulation experiments (needed in parameter scans) and steady state experiments. In addition to new simulation types, another important extension is the ability to consume experimental data and directly address previously-performed simulation results. This will open the door to further analyses such as parameter fitting and optimization tasks. Eventually, this will make SED-ML the format of choice for a compact but comprehensive description of simulation experiments, allowing for the seamless exchange of model, experimental data and simulation results between software tools. We also are hopeful that SED-ML will be used by Taverna-based workflows such as those presented in [23].

## Conclusions

Reproducibility of simulation procedures is a basic requirement when working with computational biology models. SED-ML provides structures to describe simulation procedures and allows to reproduce them. The provision of a SED-ML file together with publicly available models simplifies the models' reuse, as the simulation settings can be directly loaded into the simulation software. Together with SBML and SBGN to describe and represent the models, SED-ML is a new cornerstone of the edifice enabling to completely encode a computational systems biology project. Since SED-ML is independent of particular model formats, we believe its use will also play a role in bridging different communities towards integrative systems biology.

## Methods

### SED-ML Community development

SED-ML is a community effort that has been developed in cooperation with several modeling and simulation groups in computational systems biology. The development of SED-ML was begun at the same time as MIASE and KiSAO during a PhD visit by DW in the group of NLN. The SED-ML project was first presented publicly at the 12^th ^SBML Forum Meeting in 2007 and its main structure outlined at both the super-hackathon "Standards and Ontologies for Systems Biology" in 2008 and the combined "CellML-SBGN-SBO-BioPAX-MIASE workshop" in 2009. Since then SED-ML has been developed in collaboration with the communities forming the "computational modeling in biology network" (COMBINE, http://co.mbine.org/). Besides dedicated sessions at various meetings, the development of SED-ML benefits from community interactions on the sed-ml-discuss mailing list (https://lists.sourceforge.net/lists/listinfo/sed-ml-discuss/). Every update in the language, as well as current issues and proposals for language extensions are discussed and voted on in an open forum. The specification development, as well as versions of the UML diagrams and the XML Schema are available from the SED-ML website. The community can also make use of a tracker to report bugs in the language or its implementation. The first official version of the SED-ML specification was published in March 2011. Since then, the community has elected editors to coordinate SED-ML development. The SED-ML editorial board consists of five editors and one editorial advisor. Editors were elected for a duration of two to four years and will be replaced accordingly.

### Language specification

SED-ML is described in full detail in the specification document, "Simulation Experiment Description Markup Language (SED-ML): Level 1 Version 1" published in Nature Preceedings in March 2011 [24] and available from the SED-ML website. The specification describes the language and also outlines the typical workflow of creating a SED-ML document; examples show the use of SED-ML with existing models. The SED-ML Level 1 Version 1 specification document is the normative document and an XML Schema and UML diagram are provided as aids for tool developers and SED-ML users. In SED-ML, major language revisions containing substantial changes result in a new "level" while minor revisions containing corrections and refinements of SED-ML elements lead to forthcoming "versions" [24].

SED-ML documents can be validated against the SED-ML XML schema. XML Schema http://www.w3.org/XML/Schema is a W3C standard for describing the structure and content of an XML document. Although the XML Schema describes the structure of SED-ML, some language restrictions described in the normative SED-ML specification document cannot be encoded in XML Schema due to its limited rule constructs. We also provide a UML representation of the language to facilitate its understanding. However, the UML diagrams shown in the SED-ML specification document only support the written text. They do not fully express the constraints of the language.

### Interaction with existing standards and technologies

SED-ML re-uses existing standards, conventions and ontologies wherever possible in order to avoid duplication of effort. SED-ML encodes any pre-processing applied to the computational model, as well as post processing applied to the raw simulation results data before output, using MathML 2.0. MathML is an international standard for encoding mathematical expressions in XML. It is also used as a representation of mathematical expressions in other formats, such as SBML and CellML, two of the model representation languages supported by SED-ML. In order to identify nodes and attributes within the XML representations of biological models, SED-ML uses XPath, a language for finding information in an XML document [8]. To identify precisely the type of simulation algorithm in the simulation experiment, SED-ML uses KiSAO [9]. Tools can for instance, use this information to differentiate whether stochastic traces or continuous simulations are requested, or to relate simulation algorithms and substitute one integration method with an equivalent one. Tools can also retrieve the parameters necessary in the configuration of an algorithm, for instance, to automatically generate the corresponding graphical interface. SED-ML is now a core standard of COMBINE, and as such we will seek to keep the maximum interoperability with other standards in computational systems biology.

## Authors' contributions

DW and NL initiated the project. All authors participated in the discussions leading to the structure of SED-ML. DW, RA, FB and NL developed the first specification of the language. All authors participated to and approved the final manuscript's preparation.

## Supplementary Material

Additional file 1 **SED-ML examples file**. Repressilator simulation described in SED-ML. Click here for file
